# Venous gas embolism: An unusual complication of laparoscopic cholecystectomy

**DOI:** 10.4103/0972-9941.55105

**Published:** 2009

**Authors:** Tim N Wenham, Donald Graham

**Affiliations:** Department of anaesthetics, Barnsley Hospital NHS Foundation Trust, Gawber Road, Barnsley, S75 2PS, England; 1 Doncaster Royal Infirmary, Armthorpe Road, Doncaster DN2 5LT, England

**Keywords:** CO_2_ embolism, laparoscopic cholecystectomy, millwheel murmur, venous gas embolism

## Abstract

Venous gas embolism (VGE) is a rare but potentially lethal complication of many forms of surgery, especially posterior fossa neurosurgery where the incidence is reported to be up to 80% - it can also occur in laparoscopic surgery. It usually occurs early in the procedure during insufflation of the abdomen. Rapid entry or large volumes of gas entering the venous circulation initiate a predictable chain of pathophysiological events which may continue to cardiovascular collapse. Arterial hypoxaemia, hypercapnia, decreased end-tidal CO_2_, arrhythmias, myocardial ischaemia and elevated central venous and pulmonary arterial pressures can occur. The management of VGE relies on a high index of suspicion and close liaison between anaesthetist, surgeon and theatre staff. The authors present a case of venous gas embolism (VGE) during laparoscopic cholecystectomy (LC) which presented without many of the usual clinical features and was diagnosed by auscultation of a millwheel murmur.

## INTRODUCTION

Venous gas embolism (VGE) is a rare but potentially lethal complication of many forms of surgery, including laparoscopic surgery. Rapid entry or large volumes of gas entering the venous circulation initiate a predictable chain of pathophysiological events which may continue to cardiovascular collapse. Arterial hypoxaemia, hypercapnia, decreased end-tidal CO_2_, arrhythmias, myocardial ischaemia and elevated central venous and pulmonary arterial pressures can occur. The authors present a case of venous gas embolism (VGE) during laparoscopic cholecystectomy (LC) which presented without many of the usual clinical features and was diagnosed by auscultation of a millwheel murmur.

## CASE REPORT

A 55-year-old woman underwent elective LC for symptomatic cholelithiasis. Although moderately overweight, she was otherwise fit and well, took no medications and had undergone uneventful general anaesthesia in the past. She was a non-smoker. The operation was performed under general anaesthesia using fentanyl, propofol and vecuronium for induction, 40% O_2_, nitrous oxide and isoflurane with intermittent positive pressure ventilation for maintenance and bolus intravenous morphine for analgesia. After 80 minutes of surgery her oxygen saturations fell from 98% to 94% and systolic arterial pressure dropped from 140 to 90 mmHg. End tidal CO_2_ (ETCO_2_) was unchanged, airway pressures and ECG trace remained normal and there were no problems with the anaesthetic equipment or breathing circuit. The surgeon confirmed that there was no evidence of intra-abdominal haemorrhage. Auscultation of the lung fields was normal, but normal heart sounds were replaced by the loud splashing sound of a millwheel murmur. The diagnosis of a VGE was made and the surgery temporarily halted and inspired oxygen concentration increased to 100%. The patient's observations returned to normal within five minutes and the operation then completed uneventfully. She was discharged form hospital three days later without any adverse sequelae.

## DISCUSSION

In recent years laparoscopic surgery has become routine, aiming to minimise the trauma of surgery with the potential benefits of reduced post-operative pain, shorter hospital stay, more rapid return to daily activities and significant cost savings.[1,2] These advantages, however, must be weighed against the risks of a new range of complications including the cardiopulmonary effects of pneumoperitoneum coupled with the often extremes of patient positioning, systemic carbon dioxide absorption, extraperitoneal gas insufflation and venous gas embolism.[2]

VGE is a rare but potentially lethal complication of many forms of surgery, especially posterior fossa neurosurgery where the incidence is reported to be up to 80% - it can also occur in laparoscopic surgery.[3] It usually occurs early in the procedure during insufflation of the abdomen,[4] but may, as in this case, happen later. Rapid entry or large volumes of gas entering the venous circulation initiate a predictable chain of pathophysiological events. Migration of the gas emboli into the pulmonary circulation increases pulmonary arterial pressure and increases resistance to right ventricular outflow-this causes decreased pulmonary venous return, decreased left ventricular preload and decreased cardiac output which may continue to cardiovascular collapse. Volumes of gas up to 50 ml may cause few sequelae but large volumes (50–300mls) may cause a variable response from mild hypotension to death. The ventilation/perfusion mismatch and alteration in the lung vessel resistance generates an intrapulmonary right-to-left shunt and increased alveolar dead space giving rise to arterial hypoxaemia, hypercapnia and decreased ETCO_2_.[5] Arrhythmias, myocardial ischaemia and elevated central venous and pulmonary arterial pressures may also occur.[6] Our patient developed hypoxia and hypotension but without a change in ETCO_2_ or other signs despite a millwheel murmur being considered a late sign of venous gas embolism.[3] Fortunately her signs reversed rapidly - presumably due to the rapid absorption of the CO_2_ - and she suffered no ill effects from the VGE. The management of VGE relies on a high index of suspicion and close liaison between anaesthetist, surgeon and theatre staff.[2,6] If the diagnosis is suspected during laparoscopic surgery, the procedure should be halted and the pneumoperitoneum released whilst flooding the abdominal cavity with irrigation fluid. The angle of the table should be altered to ensure the heart is above the level of the operation site.[7] Increasing the FiO_2_ (to 100% if necessary) may reduce the size of the gas bubbles and a central venous catheter can allow aspiration of gas from the right heart on occasion; this is aided by turning the patient into the left lateral position.[4,5] Volume expansion and inotropes may be necessary to counteract the systemic hypotension. Occasionally hyperbaric oxygen therapy may be needed.[7]

In summary, we present a case of venous gas embolism during an otherwise routine laparoscopic cholecystectomy, which presented with sudden hypoxia and hypotension and was diagnosed by clinical signs alone. It serves to remind clinicians of this potentially lethal complication of laparoscopic surgery and of an uncommon physical sign.

## References

[1] Chui PT, Gin T, Oh TE (1993). Anaesthesia for laparoscopic general surgery. Anaesth Intensive Care.

[2] Leonard IE, Cunningham AJ (2002). Anaesthetic considerations for laparoscopic cholecystectomy. Best Pract Res Clin Anaesthesiol.

[3] Wittenberg AG, Richard AJ, Conrad SA (2006). Venous air embolism.

[4] McIndoe A, Allman KG, Wilson IH (2001). Anaesthetic emergencies. Oxford Handbook of Anaesthesia.

[5] Muth CM, Shank ES (2000). Gas embolism. N Engl J Med.

[6] Wahba RW, Tessler MJ, Kleiman SJ (1996). Acute ventilatory complications during laparoscopic upper abdominal surgery. Can J Anaesth.

[7] Gildenberg PL, O'Brien RP, Britt WJ, Frost EA (1981). The efficacy of doppler monitoring for the detection of venous air embolism. J Neurosurg.

